# Microbiota signatures and mucosal healing in the use of enteral nutrition therapy *v*. corticosteroids for the treatment of children with Crohn’s disease: a systematic review and meta-analysis

**DOI:** 10.1017/S0007114523000405

**Published:** 2023-10-28

**Authors:** Zhaolu Ding, Kiran Ninan, Bradley C. Johnston, Paul Moayyedi, Mary Sherlock, Mary Zachos

**Affiliations:** 1 Department of Health Research Methods, Evidence and Impact, McMaster University, Hamilton, ON, Canada; 2 Department of Nutrition, College of Agriculture and Life Sciences, Texas A&M University, College Station, TX, USA; 3 Department of Epidemiology and Biostatistics, School of Public Health, Texas A&M University, College Station, TX, USA; 4 Department of Medicine, Division of Gastroenterology, McMaster University, Hamilton, ON, Canada; 5 Division of Gastroenterology, Hepatology and Nutrition, Department of Pediatrics, McMaster University, Hamilton, ON L8S 4K1, Canada

**Keywords:** Systematic review, Child, Inflammatory bowel disease, Enteral nutrition, Corticosteroids, Effectiveness

## Abstract

Corticosteroids (CS) and exclusive and partial enteral nutrition (EEN and PEN) are effective therapies in paediatric Crohn’s disease (CD). This systematic review of randomised controlled trials (RCT) and cohort studies analyses the impact of EEN/PEN *v*. CS on intestinal microbiota, mucosal healing as well as other clinically important outcomes, including clinical remission, relapse, adherence, adverse events and health-related quality of life (HRQL) in paediatric CD. Three RCT (*n* 76) and sixteen cohort studies (*n* 1104) compared EEN *v*. CS. With limited available data (one RCT), the effect on intestinal microbiome indicated a trend towards EEN regarding Shannon diversity. Based on two RCT, EEN achieved higher mucosal healing than CS (risk ratio (RR) 2·36, 95 % CI (1·22, 4·57), low certainty). Compared with CS, patients on EEN were less likely to experience adverse events based on two RCT (RR 0·32, 95 % CI (0·13, 0·80), low certainty). For HRQL, there was a trend in favour of CS based on data from two published abstracts of cohort studies. Based on thirteen cohort studies, EEN achieved higher clinical remission than CS (RR 1·18, 95 % CI (1·02, 1·38), very low certainty). Studies also reported no important differences in relapse and adherence. Compared with CS, EEN may improve mucosal healing with fewer adverse events based on RCT data. While limited data indicate the need for further trials, this is the first systematic review to comprehensively summarise the data on intestinal microbiome, mucosal healing and HRQOL when comparing enteral nutrition and CS in paediatric CD.

Inflammatory bowel disease (IBD) is a chronic relapsing inflammatory condition of the digestive tract^(1,2)^. As a type of IBD, Crohn’s disease (CD) has no proven cure and can impact proper digestion and absorption, which can result in malnutrition in children^(1–3)^. Exclusive enteral nutrition (EEN) and corticosteroids (CS) are both proven to be effective therapies for the induction of remission in paediatric CD^(4–6)^. The use of CS has raised concerns due to possible side effects, including reduced bone density and growth delay^(7)^. Given the safety concerns, there has been an increasing interest in the use of EEN to induce remission of active CD. EEN may have a profound impact on microbiota diversity and inflammation marker levels^(8–10)^. However, conflicting results exist in previous studies^(11–13)^. Furthermore, the implementation of EEN is challenging as it commonly requires the use of a nasogastric feeding tube for 6–8 weeks along with avoidance of other food intake, which may reduce the compliance of the child and family^(14,15)^. To improve adherence, more studies have focused on partial enteral nutrition (PEN), which allows children to take some whole food alongside an enteral formula^(16)^. Recent studies in adults and children reported that PEN could be as effective as EEN in inducing clinical and endoscopic remission in children with active CD, and PEN was better tolerated by paediatric patients^(14,16–19)^.

The mechanism underlying the clinical effectiveness of EEN and PEN in paediatric IBD patients remains unclear. One hypothesis is that EEN and PEN may induce changes in the faecal microbiome and this could promote remission^(14)^. Recent data in humans illustrate that dysbiosis plays an important role in the development of IBD^(1)^, and enteral nutrition may have a profound impact on the microbiota diversity^(8–10)^. A previous systematic review compared the effectiveness of EEN and PEN *v*. CS, but the authors mostly focused on the clinical remission of CD^(7)^. In addition to intestinal microbiota, more recently, mucosal healing is an outcome that is gaining acceptance as a recommended measure of disease activity in CD^(6,20)^. Two systematic reviews assessed mucosal healing between EEN and CS in the paediatric population but did not consider the effect of EEN or PEN on intestinal microbiota^(21,22)^.

We conducted a systematic review and meta-analysis to determine the impact of both EEN and PEN *v*. CS in children with active luminal CD on intestinal microbiota, mucosal healing, clinical remission, relapse of active disease, post-treatment weight, faecal calprotectin (FC), health-related quality of life (HRQL), adherence to the assigned intervention and adverse events up to 12 months following initial treatment.

## Materials and methods

### Study selection and patient population (inclusion and exclusion criteria)

Our study protocol was registered on PROSPERO (CRD42021254082). We considered both randomised controlled trials (RCT) and cohort studies in children (≤18 years of age) with newly diagnosed or active luminal CD according to the Pediatric Crohn’s Disease Activity Index (PCDAI), defined as a score >10, or alternatively, other clearly defined definitions of newly diagnosed or active CD by investigators. Studies that compared the administration of any type of enteral nutrition (i.e. elemental, semi-elemental or polymeric) to CS (e.g. methylprednisolone, prednisone or hydrocortisone) were considered for inclusion. Randomised trials and cohort studies were analysed separately. We excluded the following types of studies: trials allowing oral intake other than clear liquids in EEN treatment, trials allowing co-interventions with antibiotics and having outcomes of microbiota analysis and trials not defining CD activity and remission.

### Data sources and search strategy

We searched the following five databases from inception until 3 February 2021: Medline, Embase, Cumulative Index to Nursing and Allied Health Literature (CINAHL), Cochrane Central Register of Controlled Trials (CENTRAL) and Clinicaltrials.gov. The references of eligible studies and review articles were searched to identify additional studies. Abstracts or posters without published full-text articles were excluded as the preliminary results often differ from final published reports^(23)^. For our target outcomes, authors were contacted for additional unpublished results, including missing outcome data. Our review had no language restrictions. Google Translate tool was used to translate articles written in non-English languages. If further clarification was required, we considered contacting translators/authors.

### Outcome measures

Our primary outcomes included intestinal microbiome signatures (e.g. sequencing with 16S ribosomal RNA gene) and mucosal healing rate (endoscopy score)^(4,8–13)^. Data on *α*-diversity metrics (e.g. relative abundance and Shannon diversity index) and *β*-diversity metrics (e.g. Bray–Curtis index to visualise clustering) were also collected for our outcome of microbiome signatures^(24)^. Shannon diversity index, clustering and relative abundance of bacterial genera if available were described as continuous variables. Mucosal healing was defined as complete endoscopic remission using the Simple Endoscopic Score for Crohn Disease (SES-CD) of 0^(25)^. When SES-CD scores were not reported, other clear definitions for mucosal healing were also considered (e.g. the Crohn’s Disease Endoscopic Index of Severity less than 3 points or a drop of >70 % at follow-up endoscopy)^(8,26)^. Our primary outcomes were assessed at 4–12 weeks after therapy initiation.

Our secondary outcomes included clinical remission (4–12 weeks after induction therapy) and clinical relapse rate (at a 6–12-month time-point). Remission and relapse were measured using the PCDAI score (remission was defined as <15 points, or <7·5 points without the height component of the index) or using other clearly defined author definitions (e.g. short PCDAI, abbreviated PCDAI and Lloyd-Still disease activity index)^(27–30)^. Clinical relapse was defined as the occurrence or worsening of symptoms accompanied by a PCDAI score > 10 points in a patient who had previously reached clinical remission^(31)^. Other secondary outcomes included nutritional status (i.e. weight in both kg and *Z*-score measurements), FC level (i.e. a biochemical marker of inflammation to implicate disease activity), adherence (i.e. withdrawal rates), adverse events and HRQL (e.g. IMPACT I–III questionnaire or other validated health status measurements) at 4–12 weeks after induction therapy^(7,32–37)^.

### Data screening (eligibility assessment) and data extraction

Titles and abstracts were independently screened by two reviewers. If inclusion criteria were met, publications were exported, screened and carried onto independent full-text screening. Discrepancies between reviewers on inclusion and exclusion decisions were resolved among themselves, and a third reviewer was involved if consensus was not reached. A piloted data collection form was used to independently extract data and assess the risk of bias (RoB) in duplicate. Data were extracted for study population characteristics, study design details, information on administration or exposure to EEN/PEN and CS, and eligible outcomes.

### Quality assessment

Two reviewers independently appraised the RoB using the Cochrane RoB tool for randomised trials (RoB 2.0)^(38)^, while the RoB for non-randomised studies of interventions (ROBINS-I) tool was used to assess cohort studies^(39)^. Overall ratings of ‘low’, ‘some concerns’ or ‘high’ were determined for each domain within the RoB 2.0 tool. Ratings of ‘low’, ‘moderate’, ‘serious’ or ‘critical’ were determined for each domain within the ROBINS-I tool. We resolved any discrepancies through discussion between the two reviewers and, when necessary, through consultation with a third senior methodologist.

### Data synthesis

We analysed aggregated data through quantitative synthesis. A random effects meta-analysis was performed due to potential heterogeneity between studies. The *I*^2^ statistic and inconsistency between studies using forest plots were used to assess heterogeneity^(40)^.

Data permitting, for cohort studies, we planned to pool adjusted and unadjusted effect sizes separately. For dichotomous outcomes, pooled risk ratios (RR) and 95 % CI were calculated (e.g. mucosal healing, clinical remission, relapse, adherence and adverse events). For continuous outcomes (e.g. microbiota diversity, bacterial abundance, FC level, HRQL score and weight), we pooled mean difference (MD) with a standard deviation or standardised mean differences (SMD) with corresponding 95 % CI. Cohen’s D scores, were used to determine the effect of SMD estimates^(41)^.

As an a priori decision, subgroup analyses were planned for the effect of: (1) EEN *v*. CS and PEN *v*. CS separately, (2) mild to moderate CD *v*. severe CD and (3) newly diagnosed CD *v*. all active CD (including previously diagnosed patients) as outcomes may differ based on previous studies^(4,8,14,42,43)^. Sensitivity analyses removing studies that are high RoB studies for each outcome were also considered. Publication bias was considered using funnel plots if there were >10 included studies for an outcome^(44,45)^. All analyses were performed using Review Manager (RevMan) Version 5.3 and Stata 16.0.

### Assessment of certainty of evidence

The Grading of Recommendations Assessment, Development and Evaluation (GRADE) tool was used to assess the certainty of evidence for the included outcomes^(46)^. Two review authors independently assessed the certainty of evidence as high, moderate, low or very low using the GRADE approach, which included assessments of RoB, inconsistency, imprecision, indirectness and publication bias.

## Results

### Characteristics and risk of bias of included studies

**Fig. 1.**
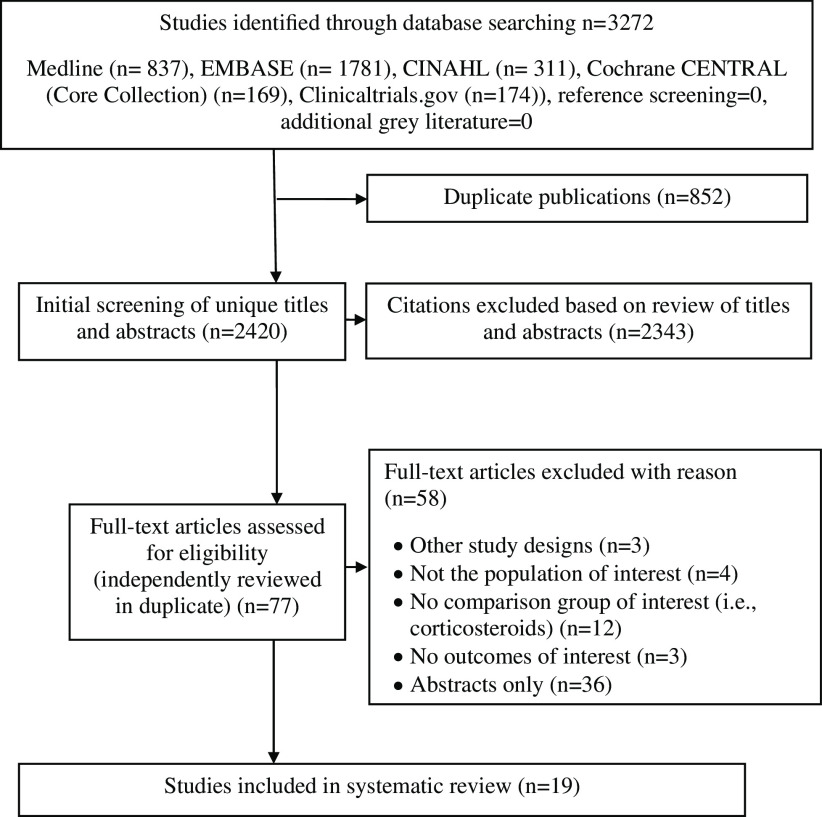
Flow diagram of the study selection process.

Our search (online Supplementary Table 1) retrieved a total of 3272 articles (Fig. 1). After excluding duplicates, we screened 2420 titles and abstracts and reviewed seventy-seven full-text articles for potential eligibility (Fig. 1). Details on important excluded studies are available in online Supplementary Table 2. A total of nineteen studies on patients with CD were included in our systematic review (Table 1). Three studies were RCT (*n* 76) that assigned participants to receive EN or CS, while the remaining sixteen studies (*n* 1104) were cohort studies that observed the effect of EN *v*. CS (five of these were prospective while eleven were retrospective) (Table 1). All nineteen studies considered the use of EEN, while CS type and dosage varied (Table 1). None of nineteen studies reported on PEN *v*. CS.

**Table 1.** Characteristics of included studies

Study, year	Study design	Indication	Follow-up period(s)	Intervention	Control	Outcome and description
Hart *et al*., 2020^(4)^	Prospective cohort	Active CD patients	8 weeks	EEN (polymeric formula) administered through a nasogastric tube for 8 weeks	Methylprednisolone (1 mg/kg per d, with a maximum dose of 40 mg/d). Once symptoms improved, patients were transitioned to oral CS and discharged home, followed by a progressive wean by 5 mg/week	• Microbiota changes, including Shannon diversity, *β*-diversity metrics, and bacteria composition • Clinical remission was defined as a PCDAI score <10
Scarpato *et al.*, 2020^(31)^	Retrospective cohort	Active CD patients (mild, moderate and severe CD)	8 weeks and 1 year	EEN (polymeric formula) administered orally or through a nasogastric tube for 8 weeks followed by a gradual introduction of foods during the subsequent 4 weeks	Oral methylprednisolone (1 mg/kg per d with a maximum dose of 40 mg/d) for 4 weeks, followed by a gradual tapering off by week 11	• Clinical remission was defined as a PCDAI score <10 with the absence of symptoms • Relapse at 12 months was defined as the occurrence or worsening of symptoms accompanied by a PCDAI score >10, in patients who had already reached clinical remission • Faecal calprotectin was measured using laboratory parameters • Weight after induction therapy (*Z*-score)
Pigneur *et al.*, 2019^(8)^	RCT	Newly diagnosed CD patients	8 weeks	EEN (formula not specified) delivered orally or by tube feeding for 8 weeks	Prednisone (1 mg/kg per d with a maximum dose of 60 mg/d) for 4 weeks, followed by tapering	• Microbiota changes, including Shannon diversity, *β*-diversity metrics, and bacteria composition • Mucosal healing was defined as CDEIS < 3 points or a drop of >70 % at follow-up endoscopy compared with the initial diagnostic endoscopy • Clinical remission was defined as an HBI < 5
Kang *et al*., 2019^(51)^	Retrospective cohort	Newly diagnosed CD patients	8 weeks	EEN (polymeric formula) administered orally for 8 weeks	Prednisone (1 mg/kg per d) for 4 weeks and had been weaned over a subsequent 2– 4 weeks	• Clinical remission was defined as a PCDAI score <10
Cohen-Dolev *et al*., 2018^(49)^	Prospective cohort	Newly diagnosed CD patients (mild and moderate CD only)	8, 12, 78, and 104 weeks	EEN (any formula) provided orally or by a nasogastric tube for 6–8 weeks	Prednisone or methylprednisolone (1–1·5 mg/kg per d) to be tapered by Week 11	• Clinical remission was defined as a PCDAI score <10
Lafferty *et al*., 2017^(53)^	Retrospective cohort	Newly diagnosed CD patients (mild, moderate and severe CD)	8 and 52 weeks	EEN (polymeric or elemental) administered either orally or via a feeding tube for 6–8 weeks	Prednisolone (1 mg/kg per d with a maximum dose of 40 mg/d) for 4 weeks, followed by a weekly 5 mg wean over a subsequent 7 weeks	• Clinical remission was defined as a PCDAI score <10 • Weight after induction therapy (Z-score) • Relapse at 12 months was defined as an increase in disease activity necessitating a repeat course of EEN or CS, an escalation of medical treatment or surgery
Connors *et al*., 2017^(50)^	Retrospective cohort	Newly diagnosed CD patients (mild, moderate and severe CD)	8 weeks and follow-up at 6 years	EEN (formula not specified) administered via nasogastric tube and treated for 8–16 weeks	Prednisone (dose not specified) for 4–12 weeks	• Clinical remission was defined as a PCDAI score <7·5
Hradsky *et al.*, 2016^(57)^	Retrospective cohort	Newly diagnosed CD patients	Week 6–12 and 40 months	EEN (any polymeric enteral formula) delivered orally or through a nasogastric tube for 6–10 weeks	Prednisolone (1–2 mg/kg per d, up to 40 mg/d and exceptionally 60 mg/d) for approximately 8 weeks with slow tapering	• Weight after induction therapy (*Z*-score)
Luo *et al*., 2015^(58)^	Retrospective cohort	Newly diagnosed CD patients (mild and moderate CD only)	9·6 weeks	EEN (polymeric formula) administered orally for 8 weeks	Prednisone/hydrocortisone for 8 weeks	• Clinical remission was defined as a PCDAI score <10
Hojsak *et al*., 2014^(43)^	Retrospective cohort	Active CD patients (mild, moderate and severe CD)	12 months	EEN (polymeric formula) administered orally or through a nasogastric tube for 6–8 weeks	‘Conventional CS’ was used as remission induction therapy	• Clinical remission was defined as a PCDAI score <10 • Relapse at 12 months was defined as a PCDAI > 10 and need for the use of remission induction therapy
Levine *et al*., 2014^(54)^	Prospective cohort	Newly diagnosed CD patients (mild and moderate CD only)	8, 12 and 52 weeks	EEN therapy group was given polymeric formula for 6–8 weeks	Prednisone (1–2 mg/kg, with a maximum of 60 mg/d)	• Clinical remission was defined as a PCDAI score <10 or <7·5 • Faecal calprotectin was measured using calprotectin assay kits
Soo *et al.*, 2013^(56)^	Retrospective cohort	Newly diagnosed CD patients (mild, moderate and severe CD)	6–8 weeks and 12 months	EEN (polymeric or semi-elemental formula) for 6 weeks and then partially over the next 2 weeks	Prednisone (1 mg/kg per d, with a maximum dose of 50 mg/d) for 4 weeks and then weaned over the next 6–8 weeks	• Clinical remission was defined as a PCDAI score <10 • Relapse at 12 months was defined as a PCDAI score >10 on a subsequent visit after achieving remission
Kierkus *et al.*, 2013^(52)^	Prospective cohort	Active CD patients (moderate and severe CD only)	8 and 52 weeks	EEN (formula not specified) provided orally or by a nasogastric tube for 6 weeks	‘Conventional steroid therapy’	• Clinical remission was defined as a PCDAI score <10 • Weight after induction therapy (kg)
Lambert *et al*., 2012^(29)^	Retrospective cohort	Newly diagnosed CD patients	6 months, 6–12 months and 12–24 months following diagnosis	EEN (polymeric formula) administered for 6–8 weeks, after completion of EEN a normal diet was reintroduced gradually	Prednisone as sole therapy for induction was considered	• Relapse at 12 months was defined as an increase in disease activity necessitating a change in management
Borrelli *et al.*, 2006^(42)^	RCT	Newly diagnosed CD patients (moderate and severe CD only)	10 weeks	EEN (polymeric diet), administered orally or through a nasogastric tube for 10 weeks	Methylprednisolone (1·6 mg/kg per d, with a maximum allowed dose of 60 mg/d) for 4 weeks, followed by a 6-week tapering course until a dose between 5 and 10 mg/d was reached	• Mucosal healing was defined as a decrease in both endoscopic and histologic scores by 50 % or more when compared with baseline values • Clinical remission was defined as a PCDAI score <10 and absence of symptoms • Weight after induction therapy (kg)
Canani *et al*., 2006^(48)^	Retrospective cohort	Newly diagnosed CD patients	8 weeks and follow-up at 1 year	EEN (polymeric diet) administered orally, whereas the other formulas were administered through a nasogastric tube for 8 weeks	Methylprednisolone (1–2 mg/kg per d, with a maximal dose of 40 mg/d) for 4 weeks with subsequent gradual tapering over another 4 weeks	• Mucosal healing was defined as improvement in endoscopic and histological scores by a reduction ≥ 1 grade on validated endoscopic/histological tools • Clinical remission was defined as a PCDAI score <10 • Relapse at 12 months was defined as a PCDAI score >10
Terrin *et al*., 2002^(47)^	RCT	Active CD patients	8 weeks	EEN (polymeric formula) administered through a nasogastric tube for 8 weeks	Methylprednisolone (1·6 mg/kg per d) for 4 weeks and tapering for 4 more weeks	• Clinical remission was defined as a PCDAI score <10
Azcue *et al.*, 1997^(30)^	Prospective cohort	Active CD patients (moderate and severe CD only)	12 weeks	EEN (formula not specified), administered through a nasogastric tube for 5–6 weeks but discontinued after a maximum of 8 weeks	Prednisolone (1 mg/kg per d) for 1 month and then a gradual daily reduction by 5 mg/week over the next 8 weeks	• Weight after induction therapy (kg)
Papadopoulou *et al*., 1995^(28)^	Retrospective cohort	Active CD patients	22 months	EEN (elemental formula) administered orally or through a nasogastric tube for 8 weeks	Prednisone (2 mg/kg per d up to a maximum dose of 60 mg/d). The dose was reduced gradually according to the clinical response of patients	• Clinical remission was defined as a Lloyd-Still disease activity index score >80

CD, Crohn’s disease; EEN, exclusive enteral nutrition; CS, corticosteroids; PCDAI, Pediatric Crohn’s Disease Activity Index; RCT, randomised controlled trial; CDEIS, Crohn’s disease index of severity; HBI, Harvey–Bradshaw Index.

**Fig. 2.**
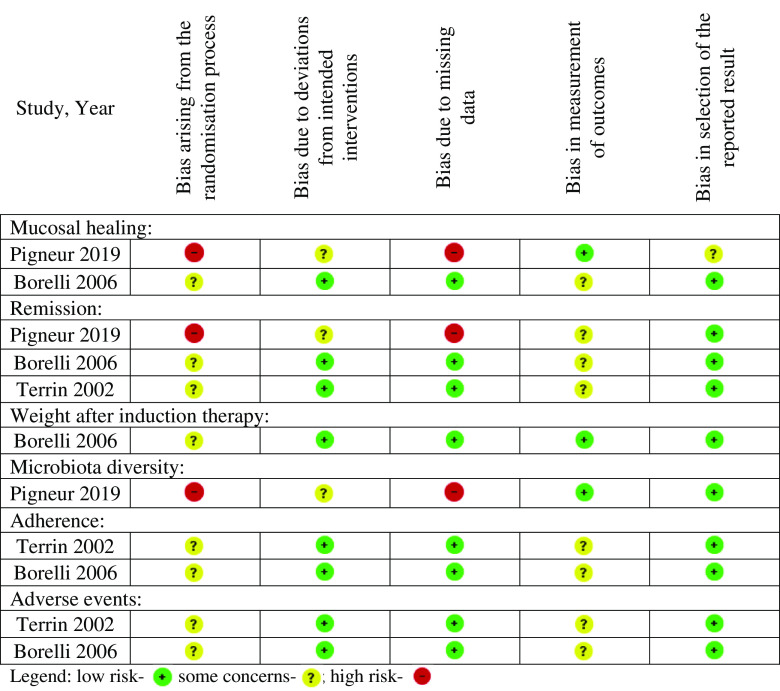
Risk of bias summary of included randomised controlled trials.

**Fig. 3.**
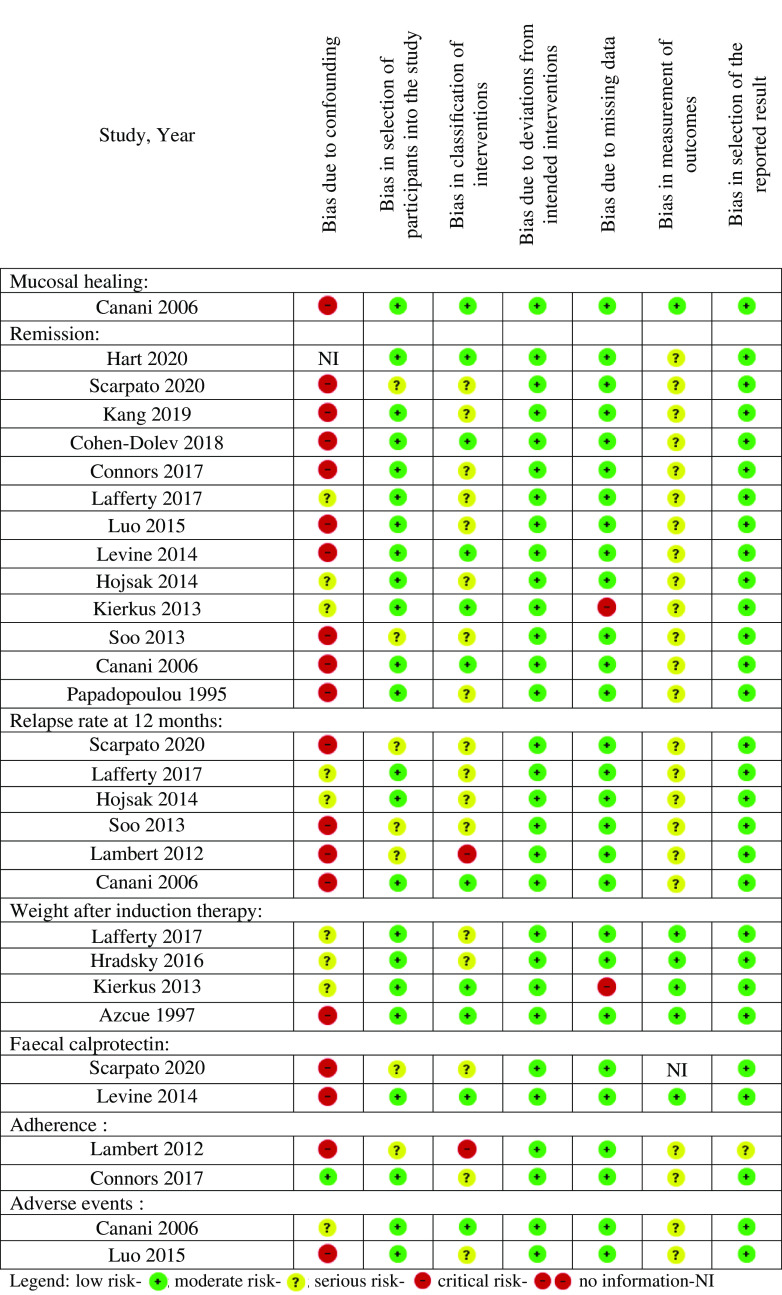
Risk of bias summary of included cohort studies.

Using the Cochrane RoB 2.0 tool for RCT, three studies had ‘some concerns’ or ‘high RoB’ for each outcome when comparing EEN *v*. CS, particularly with respect to bias in the randomisation process and bias in measurement of outcomes (Fig. 2). Similarly, sixteen cohort studies were at serious RoB for each of the outcomes due to a lack of measurement/control of important confounders (Fig. 3).

With regard to subgroup analysis, no studies reported on the use of PEN *v*. CS, or mild to moderate CD *v*. severe CD, so a priori subgroup analyses were not completed. Two RCT enrolled patients with newly diagnosed CD only^(8,42)^, while one RCT enrolled all active CD^(47)^. Furthermore, ten cohort studies included patients with newly diagnosed CD only, while the remaining six cohort studies included patients with all active CD (Table 1). Sensitivity analyses based on the RoB were not conducted based on a priori decision in the protocol as no studies had a low RoB.

### Effects of interventions

#### Microbial signatures

One RCT (*n* 19, 19 CD) and one cohort study (*n* 30, 20 CD, 10 UC) assessing EEN *v*. CS reported on stool microbial diversity and bacterial abundance^(4,8)^. In the RCT (*n* 19), Shannon diversity index, which was assessed in four patients in each group, suggested that microbial *α*-diversity tended to increase after EEN therapy (from 3·82 to 5·0), whereas the change was minimal on steroid therapy (from 5·39 to 5·75)^(8)^. The RCT also reported on *β*-diversity index based on principal components analysis of dominant microbiota composition, indicating a significant clustering before treatment and during CS or EEN treatment. Concerning bacterial abundance at genus and species levels measured at 8 weeks, both EEN (*n* 4) and CS (*n* 4) groups caused significant changes in the microbiota composition after treatment (Table 2).

**Table 2.** The results of the microbiota outcome before and after treatment in EEN and CS groups

Study	*α*-diversity index (Shannon)	*β*-diversity index	Bacterial abundance at the genus level	Bacterial abundance at the species level
Pigneur *et al*., 2019^(8)^ (RCT)	EEN (*n* 4): ↑ from 3·82 to 5·0; CS (*n* 4): ↑from 5·39 to 5·75 (minimal change)	EEN or CS: significant clustering before and during treatment (*P* = 0·049)	EEN (*n* 4): ↑ *Clostridium XIVa*; ↓ *Faecalibacterium* and *Roseburia* CS (*n* 4): ↑ *Ruminococcus* ↓ *Roseburia*	EEN (*n* 4): ↑*Clostridium symbiosum, C. ruminantium*, *Ruminococcus torques*, *Ruminococcus gnavus* and *Clostridium hathewayi* CS (*n* 4): ↑ *Bacterium M62*, *A186*, *Roseburia intestinalis*, *Eubacterium* and *Bifidobacterium bifidum*
Hart *et al*., 2020^(4)^ (Cohort)	EEN (*n* 16): ↑ CS (*n* 4): ↑	EEN (*n* 16) and CS (*n* 4): tighter and greater clustering	EEN (*n* 16): ↓ F*usobacterium*, *Escherichia/Shigella* and *Veillonella* CS (*n* 4): ↓ *Alistipes*, *Veillonella* and *Fusobacterium*	

RCT, randomised controlled trial; EEN, exclusive enteral nutrition; CS, corticosteroids.

Another cohort study with twenty CD patients reported microbiota Shannon diversity index, clustering and relative abundance but did not provide specific values for each group^(4)^. The study noted a significant increase in Shannon diversity over time after treatment (*P* = 0·006) in both EEN and CS treatments, but the increase did not differ between the groups. Based on the principal coordinates analysis for bacterial abundance, tighter clustering was observed at the end of treatment when compared with stool microbiota at baseline, independent of treatment type. Patients treated with EEN (*n* 16) showed a marked depletion in the *Fusobacterium*, *Escherichia/Shigella* and *Veillonella* genera, while patients treated with CS (*n* 4) showed reductions in the *Alistipes*, *Veillonella* and *Fusobacterium* genera.

Meta-analysis and forest plots were not generated for the two microbiome signature studies due to limited available data.

#### Mucosal healing

**Fig. 4.**
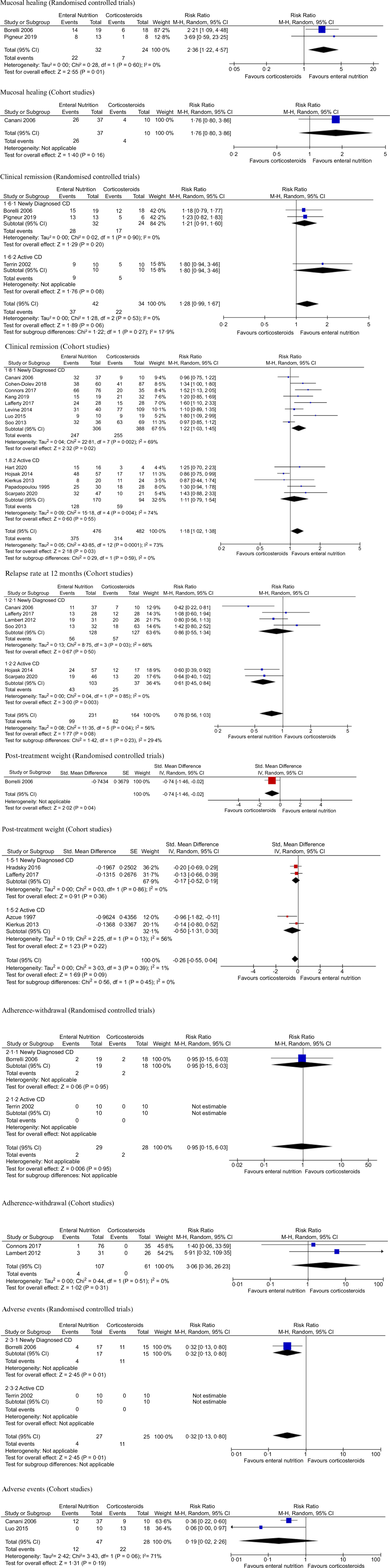
Forest plots for comparison of outcomes between enteral nutrition *v*. corticosteroids in children with Crohn’s disease (CD).

Two RCT with fifty-six participants provided data on mucosal healing^(8,42)^. We found an RR of 2·36 (95 % CI (1·22, 4·57); *I*^2^ = 0 %) (Table 3, Fig. 4). In absolute effects, forty more children had mucosal healing per 100 children receiving EEN (95 % CI, from 6 more to 100 more) (Table 3), a moderate effect size based on low certainty of evidence. Subgroup analysis could not be completed as both studies were from the newly diagnosed CD group.

**Table 3.** Summary of findings (95 % confidence intervals)

Certainty assessment							No. of patients (%)				Effect				Certainty	Importance
No. of studies	Study design	Risk of bias	Inconsistency	Indirectness	Imprecision	Other considerations	EEN		CS		Relative	95 % CI	Absolute	95 % CI		
Mucosal healing (RCT)																
2^(8,42)^	Randomised trials	Serious*	Not serious	Not serious	Serious†	None	22/32	68·8	7/24	29·2	RR 2·36	1·22, 4·57	40 more/100 (from 6 more to 100 more)		⨁⨁◯◯ Low	IMPORTANT
Mucosal healing (cohort studies)																
1^(48)^	Observational studies	Serious‡	Not serious	Not serious	Very serious§	None	26/37	70·3	4/10	40·0	RR 1·76	0·80, 3·86	30 more/100 (from 8 fewer to 100 more)		⨁◯◯◯ Very low	IMPORTANT
Remission (RCT)																
3^(8,42,47)^	Randomised trials	Serious*	Not serious	Not serious	Very serious§	None	37/42	88·1	22/34	64·7	RR 1·28	0·99, 1·67	18 more/100 (from 1 fewer to 43 more)		⨁◯◯◯ Very low	IMPORTANT
Remission (cohort studies)																
13^(4,28,31,43,48–56)^	Observational studies	Serious‡	Serious‖	Not serious	Not serious	Publication bias strongly suspected¶	375/476	78·8	314/482	65·1	RR 1·18	1·02, 1·38	12 more/100 (from 1 more to 25 more)		⨁◯◯◯ Very low	IMPORTANT
Relapse at 12 months (cohort studies)																
6^(29,31,43,48,53,56)^	Observational studies	Serious‡	Serious‖	Not serious	Very serious§	None	99/231	42·9	82/164	50·0	RR 0·76	0·56, 1·03	12 fewer/100 (from 22 fewer to 2 more)		⨁◯◯◯ Very low	IMPORTANT
Weight after induction therapy (RCT)																
1^(42)^	Randomised trials	Serious**	Not serious	Not serious	Very serious†	none	17		15		–		SMD 0·74 sd lower (1·46 lower to 0·02 lower)		⨁◯◯◯ Very low	IMPORTANT
Weight after induction therapy (cohort studies)																
4^(30,52,53,57)^	Observational studies	Serious‡	Not serious	Not serious	Very serious§	None	83		100		–		SMD 0·26 sd lower (0·55 lower to 0·04 higher)		⨁◯◯◯ Very low	IMPORTANT
Adherence withdrawal (RCT)																
2^(42,47)^	Randomised Trials	Serious*	Not Serious	Not Serious	Very Serious§	None	2/29	6·9	2/28	7·1	RR 0·95	0·15, 6·03	0 fewer/100 (from 6 fewer to 36 more)		⨁◯◯◯ Very low	IMPORTANT
Adherence withdrawal (cohort studies)																
2^(29,50)^	Observational studies	Serious‡	Not serious	Not serious	Very serious§	None	4/107	3·7	0/61	0·0	RR 3·06	0·36, 26·23	0 fewer/100 (from 0 fewer to 0 fewer)		⨁◯◯◯ Very low	IMPORTANT
Adverse events (RCT)																
2^(42,47)^	Randomised trials	Serious*	Not serious	Not serious	Serious††	None	4/27	14·8	11/25	44·0	RR 0·32	0·13, 0·80	30 fewer/100 (from 38 fewer to 9 fewer)		⨁⨁◯◯ Low	IMPORTANT
Adverse events (cohort studies)																
2^(48,58)^	Observational studies	Serious‡	Serious‡‡	Not serious	Very serious§	None	12/47	25·5	22/28	78·6	RR 0·19	0·02, 2·26	64 fewer/100 (from 77 fewer to 99 more)		⨁◯◯◯ Very low	IMPORTANT

EEN, exclusive enteral nutrition; CS, corticosteroids; RCT, randomised controlled trial; RR, risk ratio; SMD, standardised mean difference.

* Serious concerns around the randomisation process (particularly with lack of allocation concealment) and issues around blinding of the outcome assessors in studies with more weight suggest some serious risk of bias.

† With a small number of sample size or total events, fragility exists within the results. Furthermore, the optimal information size threshold is not met, and the effect estimate overlaps the GRADE recommended threshold for appreciable benefit, suggesting imprecision.

‡ When considering the included study/studies bias due to confounding, which is an important domain in the risk of bias tool, was not fully addressed. At least one important baseline confounder (e.g. disease severity, disease location, co-morbidities, concomitant medications, anthropometric measurements) was not measured or controlled for studies that hold more weight within the meta-analyses.

§ With a small number of sample size or total events, fragility exists within the results. Furthermore, CI include the possibility of a small or no effect and important benefit or harm, suggesting imprecision.

‖ There is a significant level of heterogeneity that subgroup analyses cannot explain. This suggests some serious inconsistencies exist between studies.

¶ Begg’s plot was suggestive of publication bias (*P* = 0·005).

** Serious concerns around the randomisation process (particularly with lack of allocation concealment) suggest some serious risk of bias.

†† With a small number of sample size and total events, fragility exists within the results.

‡‡ There is unexplained heterogeneity that exists. Subgroup analyses were not feasible due to a limited number of studies.

Only one retrospective cohort study with forty-seven participants reported on mucosal healing^(48)^. From this study, based on very low certainty of evidence, we found a RR of 1·76 (95 % CI (0·80, 3·86)) and a corresponding risk difference indicating that thirty more children will experience mucosal healing per 100 children receiving EEN (95 % CI from 8 fewer to 100 more) (Table 3, Fig. 4).

#### Clinical remission

Remission was assessed in three RCT^(8,42,47)^ and thirteen cohort studies^(4,28,31,43,48–56)^. When considering RCT evidence, seventy-six participants provided data. From the pooled analysis, we calculated a RR of 1·28 (95 % CI (0·99, 1·67); *I*^2^ = 0 %, very low certainty of evidence), which in absolute effects means eighteen more children had remission per 100 children receiving EEN (from 1 fewer to 43 more) (Table 3, Fig. 4).

**Fig. 5.**
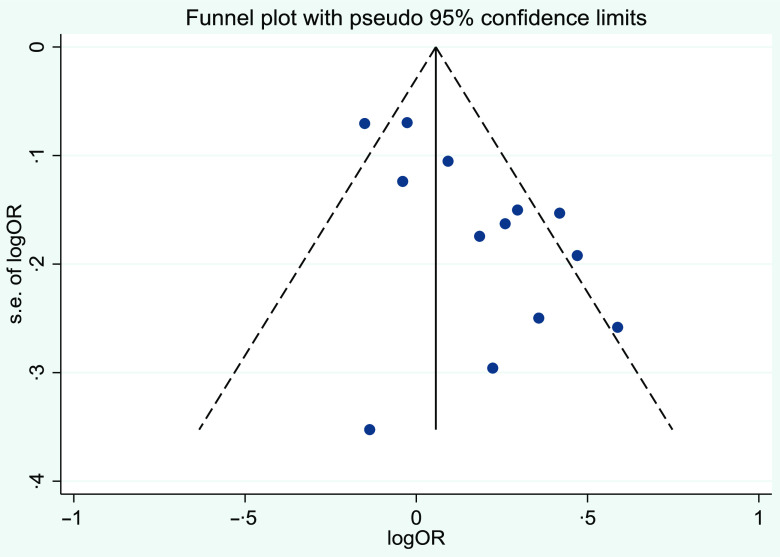
Funnel plot for cohort studies of clinical remission.

When considering cohort studies, based on thirteen studies, a total of 958 participants were included in the pooled analysis. We calculated a RR of 1·18 (95 % CI (1·02, 1·38); *I*^2^ = 73 %, very low certainty of evidence), which in absolute effects means twelve more children will experience remission per 100 children receiving EEN (from 1 more to 24 more) (Table 3). However, there was substantial heterogeneity present for this outcome (*I*^2^ = 73 %). The test of interaction for the subgroup analysis based on newly diagnosed CD *v*. all active CD was not significant in cohort studies (*P* = 0·59), and heterogeneity remained within the newly diagnosed CD group, suggesting the heterogeneity was not well explained by this subgroup analysis (Fig. 4). Furthermore, there were concerns regarding publication bias (*P* = 0·005) (Fig. 5).

#### Relapse

For relapse at 12 months, we found six cohort studies^(29,31,43,48,53,56)^ with 395 children that found an overall RR of 0·76 (95 % CI (0·56, 1·03); *I*^2^ = 56 %, very low certainty of evidence) (Fig. 4). As compared with CS, there were twelve fewer (22 fewer to 2 more) relapse events per 100 patients followed in the EEN intervention group (Table 3). Subgroup analysis for newly diagnosed *v*. all active CD found no statistically significant effect (*P* = 0·23) between the two groups. Substantial heterogeneity was still present in the newly diagnosed CD group, and significant heterogeneity for the overall effect (*I*^2^ = 56 %) was not well explained (Fig. 4). No RCT evidence was available for this outcome.

#### Nutritional status

One RCT reported on post-treatment weight for thirty-two children^(42)^. The SMD in post-treatment weight was 0·74 sd units lower in the EEN group (SMD -0·74, 95 % CI (-1·46, -0·02), very low certainty of evidence) than the CS group (Fig. 4). When the MD was described as a weighted MD, the EEN group achieved a 2·40 kg lower post-treatment weight compared with the CS group (MD -2·40, 95 % CI (-4·59, -0·21)). Among the four cohort studies with 183 children reporting on post-treatment weight^(30,52,53,57)^, we found a lower SMD of 0·26 sd units in the EEN group compared with the CS group (SMD -0·26, 95 % CI (-0·54, 0·04); *I*^2^ = 1 %, very low certainty of evidence) (Table 3). When the MD was described as a weighted MD in two cohort studies (*n* 62)^(30,52)^, the EEN group achieved a 5·20 kg lower post-treatment weight compared with CS group (MD -5·20, 95 % CI (-14·11, 3·71)). When the MD was described as a weighted MD for *Z*-score in another two cohort studies (*n* 121)^(53,57)^, the EEN group achieved 0·22 lower post-treatment weight compared with CS group (MD -0·22, 95 % CI (-0·74, 0·31)).

#### Faecal calprotectin

Two cohort studies considered our outcome measuring FC levels^(31,54)^. Meta-analyses were not feasible as data were available as medians accompanied by a range. Both studies simply reported non-significant differences in FC values at week 8 since diagnosis of CD (Levine *et al*., (1736 (617–2000) µg/g in EEN group and 558 (162–1848) µg/g in CS group)^(54)^; Scarpato *et al.*, 291·5 (15–1470) µg/g in EEN group and 435 (20–610) µg/g in CS group^(31)^). No forest plots were generated from the FC studies due to limited data.

#### Adherence (withdrawal rate)

The outcome of adherence to the intervention was reported in two RCT with fifty-seven participants^(42,47)^ and two cohort studies with 168 participants^(29,50)^. In two RCT, we calculated a RR of 0·95 (95 % CI (0·15, 6·03), very low certainty of evidence), which in absolute effects means no more (0) children had withdrawal per 100 children receiving EEN (from 6 fewer to 36 more) (Table 3). In two cohort studies, we calculated a RR of 3·06 (95 % CI (0·36, 26·23), very low certainty of evidence), which in absolute effects means no more (0) children will have withdrawal per 100 children receiving EEN as there were no events in the control group (Table 3). No significant heterogeneity was present for this outcome (*I*^2^ = 0 %). The reasons for withdrawal in EEN group were inability to introduce the formula, intolerance of the nasogastric tube feeding and development of an enterovesical fistula. The reason for two withdrawal events in the steroid therapy group was the worsening of disease activity.

#### Adverse events

We found two RCT (*n* 52)^(42,47)^ and two cohort studies (*n* 75) reported on this outcome^(48,58)^. When considering RCT evidence, we found a RR of 0·32 (95 % CI (0·13, 0·80), low certainty of evidence) (Table 3). In absolute effects, when compared with CS, there were thirty fewer (38 fewer to 9 fewer) patients with adverse events per 100 patients in the EEN group (Table 3). When considering cohort studies, as compared with CS, there was a RR of 0·19 (95 % CI (0·02, 2·26), very low certainty of evidence), which means sixty-four fewer (77 fewer to 99 more) patients with adverse events per 100 patients in the EEN group (Table 3). Significant heterogeneity for the overall effect (*I*^2^ = 71 %) was not explained, and subgroup analyses were not feasible due to a limited number of studies (Fig. 4).

Adverse events described in the EEN group are abdominal pain/discomfort, nausea, vomiting, flatulence, diarrhoea and insomnia, whereas, in the CS group, adverse events described include abdominal pain, nausea and/or vomiting, flatulence, insomnia, cushingoid appearance, acne, skin striae, hirsutism, myopathy/muscle weakness, headache, depression, hyperglycaemia and osteoporosis. No serious adverse event was reported.

#### Health-related quality of life

No studies that met our eligibility criteria reported on the HRQL outcome, and no forest plots were generated due to limited data. A list of important excluded studies (e.g. abstract only) can be found in online Supplementary Table 2. Based on the published abstracts, one prospective cohort study of thirty-one children reported a small but significant difference in generic HRQL (KIDSCREEN-10 index) between the children on CS (higher HRQL) *v*. those on EEN (MD 2·24 points, 95 % CI (0·34, 4·15))^(59)^. The MD and 95 % CI in the abstract were lower than the minimal important difference estimate of 4·53 obtained from parental ratings of KIDSCREEN-10 index^(60)^. Another prospective cohort study (*n* 64) did not find a significant difference in the disease-specific HRQL score between children receiving either EEN or CS^(61)^.

## Discussion

### Summary of main results and certainty of evidence

Our systematic review found three RCT and sixteen cohort studies having evaluated enteral nutrition in children with CD. Among two RCT (*n* 56)^(8,42)^ based on low certainty of evidence, at 4–12 weeks after induction EEN may result in an increase in mucosal healing in 40 per 100 children followed (from 6 more to 100 more) when compared with CS. Based on three RCT (*n* 76)^(8,42,47)^, eighteen more children had clinical remission per 100 children receiving EEN (from 1 fewer to 43 more), based on very low certainty of evidence. In one RCT (*n* 32)^(42)^, we found that children on EEN experienced 2·40 kg lower post-treatment weight as compared with CS alone (4·59 lower to 0·21 lower), based on very low certainty evidence. Among two RCT (*n* 52) on EEN therapy^(42,47)^, thirty fewer children per 100 followed (38 fewer to 9 fewer) were likely to experience adverse events based on low certainty evidence. On the basis of very low certainty of evidence, no significant effect on adherence outcome was detected^(42,47)^. With respect to intestinal microbial signatures described in one RCT^(8)^, a narrative synthesis was completed due to limited available data. Although the effect on the Shannon diversity seems to indicate a trend towards EEN, it is not possible to conclude the efficacy of treatment based on the very limited sample size.

When reviewing cohort studies (*n* 1104 participants), twelve more children had clinical remission per 100 children receiving EEN (from 1 more to 24 more), but the certainty of evidence is very low^(4,28,31,43,48–56)^. In addition, the evidence is very uncertain for the effect of EEN on mucosal healing^(48)^, relapse at 12 months^(29,31,43,48,53,56)^, post-treatment weight^(30,52,53,57)^, and adherence^(8,50)^, and adverse events^(48,58)^. With regard to intestinal microbial signatures, HRQL and FC^(4,31,54)^, a narrative synthesis was completed due to a lack of available data, and the potential effects were unclear.

### Strengths and limitations

Strengths of our systematic review included a comprehensive search of five databases as well as the use of internationally recognised tools to assess RoB and certainty of evidence^(38,39)^. We also considered two study designs and nine outcomes to provide a more comprehensive understanding of the literature on enteral nutrition therapy in paediatric CD. This is the first systematic review to compare stool microbiome and HRQL between enteral nutrition and CS in paediatric CD. However, limitations to the data presented are important to consider. First, for most included studies with limited sample size, especially observational studies, important baseline confounding factors such as disease severity, concomitant medications and anthropometric measurements are important to consider^(31,43,54,57,62)^. Sixteen cohort studies were at serious risk due to a lack of measurement/control of these important confounders. Therefore, the results from the cohort studies should be interpreted with caution, although underpowered and small studies should still be used as the best available evidence^(63)^. Second, our review did not address the cost-effectiveness analysis of EEN *v*. CS in patients with CD, which may have important clinical considerations when assigning patients to the induction therapy^(64)^. Finally, although the authors were contacted, we were not able to obtain complete information on microbiota signatures from two studies, which may have provided additional data for our quality assessment and meta-analysis.

### Meaning of the study and relation to previous studies

Treatments for induction of remission in children with active CD include enteral nutrition, CS and biologic agents^(3)^. Recently, enteral nutrition has been recommended as primary therapy in children with active CD due to the remission induction efficacy^(3,6)^. Similar to our study’s conclusions from RCT evidence, three previous systematic reviews determined no significant differences between EEN and CS in clinical remission in the paediatric population^(7,21,22)^. However, our conclusion based on cohort studies is different and suggests that EEN seems to be beneficial in clinical remission, but the evidence is uncertain. Similar to another systematic review, the evidence on 1-year relapse rates between EEN and CS remains uncertain but trends towards lower relapse rates in the EEN group^(21)^. In addition to clinical symptoms, therapeutic goals have changed with a recent focus on targeting objective improvement, including mucosal/histological healing^(6,20)^. Although patients treated with CS may achieve similar clinical remission and HRQL outcomes, they may fail to induce mucosal healing^(7,59,61,65)^. Similar to recent systematic reviews^(7,21,22)^, outcomes of mucosal healing based on two RCT in our review showed that children on EEN were more likely to achieve endoscopic verified mucosal healing than children administered CS. Despite low certainty of evidence, the potential advantage of enteral nutrition over CS treatment may be clinically appealing when weighing the therapeutic options for treating paediatric CD. Furthermore, recent studies indicated that EEN might have a therapeutic impact on the microbiota diversity and inflammation marker levels, although conflicting results exist among paediatric and adult studies^(8–10)^. From two related studies, we found only one RCT that reported on microbial diversity values as measured through the Shannon index in just four children in each group^(8)^. In another cohort study of twenty patients with CD, there were incomplete microbiota values with respect to microbiota diversity and bacterial abundance^(4)^. Regarding microbiota indices, sparse data and heterogeneity exist between the two studies, although the effect on the Shannon diversity index seems to indicate a trend in favour of EEN in the RCT (Table 2).

With regard to the weight changes after treatment, a previous systematic review showed that weight gain in the EEN group was higher than the CS group but was not statistically significant^(21)^. One RCT in our review reported that the post-treatment weight was lower in the EEN group *v*. the CS group^(42)^. However, weight and BMI may provide an inaccurate and misleading assessment of body composition analysis which divides the body into fat-free mass (lean mass) and fat mass. CS may lead to an increase in fat mass and a decrease in lean mass, so the misinterpretation of clinical parameters of nutrition may mask potential deficits in lean mass and malnutrition after steroid treatment^(66–69)^. For HRQL, unfortunately, no RCT or cohort studies met our eligibility criteria. While authors of the related studies were contacted for more information based on the published conference abstracts, the full-text articles with additional data were not successfully obtained to conduct a meta-analysis and generate forest plots. However, one prospective cohort study in Canada (abstract only) reported a higher generic HRQL score in the CS group compared with the EEN group and indicated a trend towards CS. To interpret the magnitude of the HRQL effect, the anchor-based minimal important difference estimate was used according to available data and published evidence^(70–72)^. Although the result was statistically significant, the MD did not meet the minimal important difference estimate^(59)^. Another prospective cohort Canadian study (abstract only) found that for children receiving either EEN or steroids for induction therapy, disease-specific HRQL scores were similar over time^(61)^. Regarding FC, there is no single standard cut-off value to implicate the presence of mucosal inflammation^(73)^. Due to this potential controversy, we did not use the dichotomous FC data for meta-analysis^(54)^. Similar to the previous systematic reviews^(7,21)^, our review indicated that children on EEN were less likely to experience adverse events when compared with steroid therapy in paediatric IBD, although the withdrawal rates do not differ between two groups. The findings may be clinically useful when assessing the risks and benefits of EEN and CS.

### Implications for practice and research

The study results may help inform clinical practices and provide guidance for the design of future research. Our findings may be useful when assessing the clinical risks and benefits of EEN and CS in children with active CD, especially for mucosal healing, clinical remission, relapse, adherence and adverse events. However, meta-analyses and determining the certainty of evidence were not feasible for the following outcomes: microbiota signatures, HRQL and FC. Our systematic review may provide valuable inferences and implications for future research areas in paediatric IBD treatment. Further RCT and cohort studies are required to better understand the applicability of EEN when considering these outcomes, especially microbiota diversity, growth parameters and FC. Crohn’s specific HRQL is also an important patient-centred metric to be evaluated and compared with anchor-based minimal important differences. Moreover, further RCT and cohort studies regarding PEN *v*. CS may expand the available literature and provide important insight into the management of paediatric IBD.

### Conclusions

Our study suggests that based on low certainty of evidence, EEN may be more beneficial than CS for mucosal healing at 4–12 weeks after induction therapy with fewer adverse events. However, the impact on clinical remission, relapse at 12 months post-induction therapy, post-treatment weight and adherence is uncertain based on very low certainty of evidence. Furthermore, the evidence on the effect of EEN compared with CS on microbiota signatures, FC and HRQL remains unclear due to limited available data, although there seems to be a trend in favour of EEN regarding gut microbiota. Additional sufficiently powered RCT are required to better assess the impact of enteral nutrition *v*. CS on paediatric CD.

## Supplementary material

For supplementary material accompanying this paper visit https://doi.org/10.1017/S0007114523000405. click here to view supplementary material

## References

[1] Pittayanon R , Lau JT , Leontiadis GI , et al. (2020) Differences in Gut microbiota in patients with *v*. without inflammatory bowel diseases: a systematic review. Gastroenterology 158, 930–946. 31812509 10.1053/j.gastro.2019.11.294

[2] Ventham NT , Kennedy NA , Nimmo ER , et al. (2013) Beyond gene discovery in inflammatory bowel disease: the emerging role of epigenetics. Gastroenterology 145, 293–308. 23751777 10.1053/j.gastro.2013.05.050 PMC3919211

[3] van Rheenen PF , Aloi M , Assa A , et al. (2021) The medical management of paediatric Crohn’s disease: an ECCO-ESPGHAN guideline update. J Crohns Colitis 15, 171–194. 10.1093/ecco-jcc/jjaa161 33026087

[4] Hart L , Farbod Y , Szamosi JC , et al. (2020) Effect of exclusive enteral nutrition and corticosteroid induction therapy on the gut microbiota of pediatric patients with inflammatory bowel disease. Nutrients 12, 1691. 32517036 10.3390/nu12061691 PMC7352362

[5] MacLellan A , Moore-Connors J , Grant S , et al. (2017) The impact of exclusive enteral nutrition (EEN) on the gut microbiome in Crohn’s disease: a review. Nutrients 9, 447. 28468301 10.3390/nu9050447 PMC5452177

[6] Ruemmele FM , Veres G , Kolho KL , et al. (2014) Consensus guidelines of ECCO/ESPGHAN on the medical management of pediatric Crohn’s disease. J Crohns Colitis 8, 1179–1207. 24909831 10.1016/j.crohns.2014.04.005

[7] Narula N , Dhillon A , Zhang D , et al. (2018) Enteral nutritional therapy for induction of remission in Crohn’s disease. Cochrane Database Syst Rev 4, CD000542. 29607496 10.1002/14651858.CD000542.pub3 PMC6494406

[8] Pigneur B , Lepage P , Mondot S , et al. (2019) Mucosal healing and bacterial composition in response to enteral nutrition *v*. steroid-based induction therapy – a randomised prospective clinical trial in children with Crohn’s disease. J Crohns Colitis 13, 846–855. 30541015 10.1093/ecco-jcc/jjy207

[9] Svolos V , Hansen R , Nichols B , et al. (2019) Treatment of active Crohn’s disease with an ordinary food-based diet that replicates exclusive enteral nutrition. Gastroenterology 156, 1354–1367. 30550821 10.1053/j.gastro.2018.12.002

[10] Schwerd T , Frivolt K , Clavel T , et al. (2016) Exclusive enteral nutrition in active pediatric Crohn disease: effects on intestinal microbiota and immune regulation. J Allergy Clin Immunol 138, 592–596. 26987574 10.1016/j.jaci.2015.12.1331

[11] Leach ST , Mitchell HM , Eng WR , et al. (2008) Sustained modulation of intestinal bacteria by exclusive enteral nutrition used to treat children with Crohn’s disease. Aliment Pharmacol Ther 28, 724–733. 19145728 10.1111/j.1365-2036.2008.03796.x

[12] Gerasimidis K , Bertz M , Hanske L , et al. (2014) Decline in presumptively protective gut bacterial species and metabolites are paradoxically associated with disease improvement in pediatric Crohn’s disease during enteral nutrition. Inflamm Bowel Dis 20, 861–871. 24651582 10.1097/MIB.0000000000000023

[13] Tang W , Huang Y , Shi P , et al. (2021) Effect of exclusive enteral nutrition on the disease process, nutrition status, and gastrointestinal microbiota for Chinese children with Crohn’s disease. JPEN J Parenter Enteral Nutr 45, 826–838. 32510616 10.1002/jpen.1938

[14] Levine A , Wine E , Assa A , et al. (2019) Crohn’s disease exclusion diet plus partial enteral nutrition induces sustained remission in a randomized controlled trial. Gastroenterology 157, 440–450. 31170412 10.1053/j.gastro.2019.04.021

[15] Lawley M , Wu JW , Navas-López VM , et al. (2018) Global variation in use of enteral nutrition for pediatric Crohn disease. J Pediatr Gastroenterol Nutr 67, e22–e29. 29543696 10.1097/MPG.0000000000001946

[16] Urlep D , Benedik E , Brecelj J , et al. (2020) Partial enteral nutrition induces clinical and endoscopic remission in active pediatric Crohn’s disease: results of a prospective cohort study. Eur J Pediatr 179, 431–438. 31781933 10.1007/s00431-019-03520-7

[17] Johnson T , Macdonald S , Hill SM , et al. (2006) Treatment of active Crohn’s disease in children using partial enteral nutrition with liquid formula: a randomised controlled trial. Gut 55, 356–361. 16162683 10.1136/gut.2004.062554 PMC1856067

[18] Sigall-Boneh R , Pfeffer-Gik T , Segal I , et al. (2014) Partial enteral nutrition with a Crohn’s disease exclusion diet is effective for induction of remission in children and young adults with Crohn’s disease. Inflamm Bowel Dis 20, 1353–1360. 24983973 10.1097/MIB.0000000000000110

[19] Sigall Boneh R , Sarbagili Shabat C , Yanai H , et al. (2017) Dietary therapy with the Crohn’s disease exclusion diet is a successful strategy for induction of remission in children and adults failing biological therapy. J Crohns Colitis 11, 1205–1212. 28525622 10.1093/ecco-jcc/jjx071

[20] Turner D , Ricciuto A , Lewis A , et al. (2021) STRIDE-II: an Update on the selecting therapeutic targets in inflammatory bowel disease (STRIDE) initiative of the international organization for the study of IBD (IOIBD): determining therapeutic goals for treat-to-target strategies in IBD. Gastroenterology 160, 1570–1583. 33359090 10.1053/j.gastro.2020.12.031

[21] Yu Y , Chen KC & Chen J (2019) Exclusive enteral nutrition *v*. corticosteroids for treatment of pediatric Crohn’s disease: a meta-analysis. World J Pediatr 15, 26–36. 30666565 10.1007/s12519-018-0204-0 PMC6394648

[22] Swaminath A , Feathers A , Ananthakrishnan AN , et al. (2017) Systematic review with meta-analysis: enteral nutrition therapy for the induction of remission in paediatric Crohn’s disease. Aliment Pharmacol Ther 46, 645–656. 28815649 10.1111/apt.14253 PMC5798240

[23] Toma M , McAlister FA , Bialy L , et al. (2006) Transition from meeting abstract to full-length journal article for randomized controlled trials. JAMA 295, 1281–1287. 16537738 10.1001/jama.295.11.1281

[24] Finotello F , Mastrorilli E & Di Camillo B (2018) Measuring the diversity of the human microbiota with targeted next-generation sequencing. Brief Bioinform 19, 679–692. 28025179 10.1093/bib/bbw119

[25] Daperno M , D’Haens G , Van Assche G , et al. (2004) Development and validation of a new, simplified endoscopic activity score for Crohn’s disease: the SES-CD. Gastrointest Endosc 60, 505–512. 15472670 10.1016/s0016-5107(04)01878-4

[26] Cellier C , Sahmoud T , Froguel E , et al. (1994) Correlations between clinical activity, endoscopic severity, and biological parameters in colonic or ileocolonic Crohn’s disease. A prospective multicentre study of 121 cases. The groupe d’etudes thérapeutiques des affections inflammatoires digestives. Gut 35, 231–235. 7508411 10.1136/gut.35.2.231 PMC1374499

[27] Turner D , Griffiths AM , Walters TD , et al. (2012) Mathematical weighting of the pediatric Crohn’s disease activity index (PCDAI) and comparison with its other short versions. Inflamm Bowel Dis 18, 55–62. 21351206 10.1002/ibd.21649

[28] Papadopoulou A , Rawashdeh MO , Brown GA , et al. (1995) Remission following an elemental diet or prednisolone in Crohn’s disease. Acta Paediatr 84, 79–83. 7734907 10.1111/j.1651-2227.1995.tb13490.x

[29] Lambert B , Lemberg DA , Leach ST , et al. (2012) Longer-term outcomes of nutritional management of Crohn’s disease in children. Dig Dis Sci 57, 2171–2177. 22661250 10.1007/s10620-012-2232-2

[30] Azcue M , Rashid M , Griffiths A , et al. (1997) Energy expenditure and body composition in children with Crohn’s disease: effect of enteral nutrition and treatment with prednisolone. Gut 41, 203–208. 9301499 10.1136/gut.41.2.203 PMC1891449

[31] Scarpato E , Strisciuglio C , Martinelli M , et al. (2020) Exclusive enteral nutrition effect on the clinical course of pediatric Crohn’s disease: a single center experience. Eur J Pediatr 179, 1925–1934. 32734315 10.1007/s00431-020-03753-x

[32] Griffiths AM , Nicholas D , Smith C , et al. (1999) Development of a quality-of-life index for pediatric inflammatory bowel disease: dealing with differences related to age and IBD type. J Pediatr Gastroenterol Nutr 28, S46–S52. 10204526 10.1097/00005176-199904001-00009

[33] Afzal NA , Van Der Zaag-Loonen HJ , Arnaud-Battandier F , et al. (2004) Improvement in quality of life of children with acute Crohn’s disease does not parallel mucosal healing after treatment with exclusive enteral nutrition. Aliment Pharmacol Ther 20, 167–172. 15233696 10.1111/j.1365-2036.2004.02002.x

[34] Loonen HJ , Grootenhuis MA , Last BF , et al. (2002) Measuring quality of life in children with inflammatory bowel disease: the impact-II (NL). Qual Life Res 11, 47–56. 12003055 10.1023/a:1014455702807

[35] Loonen HJ , Grootenhuis MA , Last BF , et al. (2002) Quality of life in paediatric inflammatory bowel disease measured by a generic and a disease-specific questionnaire. Acta Paediatr 91, 348–354. 12022311 10.1080/08035250252834049

[36] Grant A , MacIntyre B , Kappelman MD , et al. (2020) A new domain structure for the IMPACT-III health-related quality of life tool for pediatric inflammatory bowel disease. J Pediatr Gastroenterol Nutr 71, 494–500. 32960540 10.1097/MPG.0000000000002824

[37] Otley A , Smith C , Nicholas D , et al. (2002) The IMPACT questionnaire: a valid measure of health-related quality of life in pediatric inflammatory bowel disease. J Pediatr Gastroenterol Nutr 35, 557–563. 12394384 10.1097/00005176-200210000-00018

[38] Sterne JAC , Savovic J , Page MJ , et al. (2019) RoB 2: a revised tool for assessing risk of bias in randomised trials. BMJ 366, l4898. 31462531 10.1136/bmj.l4898

[39] Sterne JA , Hernan MA , Reeves BC , et al. (2016) ROBINS-I: a tool for assessing risk of bias in non-randomised studies of interventions. BMJ 355, i4919. 27733354 10.1136/bmj.i4919 PMC5062054

[40] Higgins JP , Thompson SG , Deeks JJ , et al. (2003) Measuring inconsistency in meta-analyses. BMJ 327, 557–560. 12958120 10.1136/bmj.327.7414.557 PMC192859

[41] Cohen J (1988) Statistical Power Analysis for the Behavioral Sciences, 2nd ed. Hillsdale, NJ: Lawrence Erlbaum.

[42] Borrelli O , Cordischi L , Cirulli M , et al. (2006) Polymeric diet alone *v.* corticosteroids in the treatment of active pediatric Crohn’s disease: a randomized controlled open-label trial. Clin Gastroenterol Hepatol 4, 744–753. 16682258 10.1016/j.cgh.2006.03.010

[43] Hojsak I , Pavic AM , Misak Z , et al. (2014) Risk factors for relapse and surgery rate in children with Crohn’s disease. Eur J Pediatr 173, 617–621. 24310524 10.1007/s00431-013-2230-1

[44] Sterne JA , Sutton AJ , Ioannidis JP , et al. (2011) Recommendations for examining and interpreting funnel plot asymmetry in meta-analyses of randomised controlled trials. BMJ 343, d4002. 21784880 10.1136/bmj.d4002

[45] Egger M , Davey Smith G , Schneider M , et al. (1997) Bias in meta-analysis detected by a simple, graphical test. BMJ 315, 629–634. 9310563 10.1136/bmj.315.7109.629 PMC2127453

[46] Schünemann H , Brożek J , Guyatt G , et al. (2013) GRADE Handbook for Grading Quality of Evidence and Strength of Recommendations. Updated October 2013. The GRADE Working Group. https://gdt.gradepro.org/app/handbook/handbook.html (accessed September 2022).

[47] Terrin G , Berni Canani R , Ambrosini A , et al. (2002) A semielemental diet (Pregomin) as primary therapy for inducing remission in children with active Crohn’s disease. Ital J Pediatr 28, 401–405.

[48] Berni Canani R , Terrin G , Borrelli O , et al. (2006) Short- and long-term therapeutic efficacy of nutritional therapy and corticosteroids in paediatric Crohn’s disease. Dig Liver Dis 38, 381–387. 16301010 10.1016/j.dld.2005.10.005

[49] Cohen-Dolev N , Sladek M , Hussey S , et al. (2018) Differences in outcomes over time with exclusive enteral nutrition compared with steroids in children with mild to moderate Crohn’s disease: results from the GROWTH CD study. J Crohns Colitis 12, 306–312. 29165666 10.1093/ecco-jcc/jjx150

[50] Connors J , Basseri S , Grant A , et al. (2017) Exclusive enteral nutrition therapy in paediatric Crohn’s disease results in long-term avoidance of corticosteroids: results of a propensity-score matched cohort analysis. J Crohns Colitis 11, 1063–1070. 28575325 10.1093/ecco-jcc/jjx060 PMC5881686

[51] Kang Y , Park S , Kim S , et al. (2019) Therapeutic efficacy of exclusive enteral nutrition with specific polymeric diet in pediatric Crohn’s disease. Pediatr Gastroenterol Hepatol Nutr 22, 72–79. 30671376 10.5223/pghn.2019.22.1.72 PMC6333584

[52] Kierkuś J , Szymańska S , Szczepański M , et al. (2013) The efficacy of total enteral nutrition in inducing remission and improving nutritional status in children with moderate to severe Crohn’s disease. Gastroenterol Rev 1, 57–61.

[53] Lafferty L , Tuohy M , Carey A , et al. (2017) Outcomes of exclusive enteral nutrition in paediatric Crohn’s disease. Eur J Clin Nutr 71, 185–191. 27876810 10.1038/ejcn.2016.210

[54] Levine A , Turner D , Pfeffer Gik T , et al. (2014) Comparison of outcomes parameters for induction of remission in new onset pediatric Crohn’s disease: evaluation of the porto IBD group “growth relapse and outcomes with therapy” (GROWTH CD) study. Inflamm Bowel Dis 20, 278–285. 24390062 10.1097/01.MIB.0000437735.11953.68

[55] Luo Y , Yu J , Zhao H , et al. (2015) Short-Term Efficacy of Exclusive Enteral Nutrition in Pediatric Crohn’s Disease: Practice in China. https://www.hindawi.com/journals/grp/2015/428354/ (accessed September 2022). 10.1155/2015/428354 PMC4464686 26106412

[56] Soo J , Malik BA , Turner JM , et al. (2013) Use of exclusive enteral nutrition is just as effective as corticosteroids in newly diagnosed pediatric Crohn’s disease. Dig Dis Sci 58, 3584–3591. 24026403 10.1007/s10620-013-2855-y

[57] Hradsky O , Copova I , Zarubova K , et al. (2016) Time to relapse in children with Crohn’s disease treated with azathioprine and nutritional therapy or corticosteroids. Dig Dis Sci 61, 2041–2050. 26971092 10.1007/s10620-016-4103-8

[58] Luo Y , Yu J , Zhao H , et al. (2015) Short-term efficacy of exclusive enteral nutrition in pediatric Crohn’s disease: practice in China. Gastroenterol Res Pract 2015, 428354. 26106412 10.1155/2015/428354 PMC4464686

[59] Hart L , Farbod Y , Halgren CR , et al. (2018) A153 measuring quality of life and disease activity in pediatric patients receiving induction therapy of exclusive enteral nutrition or corticosteroids for active inflammatory bowel disease. J Can Assoc Gastroenterol 1, 264–265.

[60] Hirschfeld G , von Brachel R & Thiele C (2020) Screening for health-related quality of life in children and adolescents: optimal cut points for the KIDSCREEN-10 for epidemiological studies. Qual Life Res 29, 529–536. 31620984 10.1007/s11136-019-02324-4

[61] Humphrey C , Grant AK , Walters T , et al. (2019) A260 health-related quality of life impact of steroids *v*. exclusive enteral nutrition for induction in a large Canadian pediatric IBD inception cohort. J Can Assoc Gastroenterol 2, 510–511.

[62] Piovani D , Pansieri C , Peyrin-Biroulet L , et al. (2021) Confounding and bias in observational studies in inflammatory bowel disease: a meta-epidemiological study. Aliment Pharmacol Ther 53, 712–721. 33296517 10.1111/apt.16222

[63] Guyatt GH , Mills EJ & Elbourne D (2008) In the era of systematic reviews, does the size of an individual trial still matter. PLoS Med 5, e4. 18177203 10.1371/journal.pmed.0050004 PMC2174963

[64] Tsertsvadze A , Gurung T , Court R , et al. (2015) Clinical effectiveness and cost-effectiveness of elemental nutrition for the maintenance of remission in Crohn’s disease: a systematic review and meta-analysis. Health Technol Assess 19, 1–138. 10.3310/hta19260 PMC4781042 25831484

[65] Neurath MF & Travis SP (2012) Mucosal healing in inflammatory bowel diseases: a systematic review. Gut 61, 1619–1635. 22842618 10.1136/gutjnl-2012-302830

[66] Bryant RV , Trott MJ , Bartholomeusz FD , et al. (2013) Systematic review: body composition in adults with inflammatory bowel disease. Aliment Pharmacol Ther 38, 213–225. 23763279 10.1111/apt.12372

[67] Wiskin AE , Wootton SA , Hunt TM , et al. (2011) Body composition in childhood inflammatory bowel disease. Clin Nutr 30, 112–115. 20728967 10.1016/j.clnu.2010.07.014

[68] Bin CM , Flores C , Alvares-da-Silva MR , et al. (2010) Comparison between handgrip strength, subjective global assessment, anthropometry, and biochemical markers in assessing nutritional status of patients with Crohn’s disease in clinical remission. Dig Dis Sci 55, 137–144. 19229617 10.1007/s10620-008-0692-1

[69] Sylvester FA , Leopold S , Lincoln M , et al. (2009) A 2-year longitudinal study of persistent lean tissue deficits in children with Crohn’s disease. Clin Gastroenterol Hepatol 7, 452–455. 19249399 10.1016/j.cgh.2008.12.017

[70] Norman GR , Sloan JA & Wyrwich KW (2003) Interpretation of changes in health-related quality of life: the remarkable universality of half a standard deviation. Med Care 41, 582–592. 12719681 10.1097/01.MLR.0000062554.74615.4C

[71] Ebrahim S , Vercammen K , Sivanand A , et al. (2017) Minimally important differences in patient or proxy-reported outcome studies relevant to children: a systematic review. Pediatrics 139, e20160833. 28196931 10.1542/peds.2016-0833

[72] Carrasco-Labra A , Devji T , Qasim A , et al. (2021) Minimal important difference estimates for patient-reported outcomes: a systematic survey. J Clin Epidemiol 133, 61–71. 33321175 10.1016/j.jclinepi.2020.11.024

[73] Jukic A , Bakiri L , Wagner EF , et al. (2021) Calprotectin: from biomarker to biological function. Gut 70, 1978–1988. 34145045 10.1136/gutjnl-2021-324855 PMC8458070

