# Supplementary material for: Microbiota signatures and mucosal healing in the use of enteral nutrition therapy v. corticosteroids for the treatment of children with Crohn’s disease: a systematic review and meta-analysis
Source: Br J Nutr. 2023 Feb 15;130(8):1385–402. doi: 10.1017/S0007114523000405 (PMC10511686; doi:10.1017/S0007114523000405)
Supplement: Supplementary file 1 [file S0007114523000405sup001.docx]

**APPENDICES**

**Supplementary Table 1** Search Strategies
**Supplementary Table 2** List of important excluded studies

.

**Supplementary Table 1** Search Strategies

| **Overview:** | | |
| --- | --- | --- |
| Interface: Ovid Database: **OVID Medline** Epub Ahead of Print, In-Process & Other Non-Indexed Citations, Ovid MEDLINE(R) Daily and Ovid MEDLINE(R) 1946 to Present Search Date: Feb 03, 2021 Study types: No limit on search Publication date: No limit on search | | |
| **Search Strategy:** | | |
| **#** | **Searches** | **Results** |
| 1 | exp Pediatrics/ | 59453 |
| 2 | exp Child/ | 1944820 |
| 3 | Adolescent/ | 2067662 |
| 4 | Child, Preschool/ | 933622 |
| 5 | (pediatric* or paediatric*).mp. | 426296 |
| 6 | (child* or adolescen* or teen* or youth*).mp. | 3624133 |
| 7 | 1 or 2 or 3 or 4 or 5 or 6 | 3711845 |
| 8 | Enteral Nutrition/ | 20033 |
| 9 | Enteral nutrition.mp. | 23744 |
| 10 | exp Diet Therapy/ | 55705 |
| 11 | (exclusive* adj4 (diet* or formula)).mp. | 1212 |
| 12 | (partial* adj4 (diet* or formula)).mp. | 1756 |
| 13 | ((polymeric or elemental) adj3 diet).mp. | 1034 |
| 14 | (diet* adj2 (intervention or therapy)).mp. | 71011 |
| 15 | 8 or 9 or 10 or 11 or 12 or 13 or 14 | 127640 |
| 16 | Crohn Disease/ | 39554 |
| 17 | Inflammatory Bowel Diseases/ | 23427 |
| 18 | Crohn*.mp. | 58443 |
| 19 | 16 or 17 or 18 | 73525 |
| **20** | **7 and 15 and 19** | **837** |

| **Overview:** | | |
| --- | --- | --- |
| Interface: Ovid Database: **Embase**1974 to 2021 February 03 Search Date: Feb 03, 2021 Study types: No limit on search Publication date: No limit on search | | |
| **Search Strategy:** | | |
| **#** | **Searches** | **Results** |
| 1 | exp Pediatrics/ | 110577 |
| 2 | exp Child/ | 2699271 |
| 3 | Adolescent/ | 1566705 |
| 4 | Child, Preschool/ | 370036 |
| 5 | (pediatric* or paediatric*).mp. | 660787 |
| 6 | (child* or adolescen* or teen* or youth*).mp. | 3538380 |
| 7 | 1 or 2 or 3 or 4 or 5 or 6 | 4164762 |
| 8 | Enteral Nutrition/ | 30012 |
| 9 | Enteral nutrition.mp. | 16431 |
| 10 | exp Diet Therapy/ | 356683 |
| 11 | (exclusive* adj4 (diet* or formula)).mp. | 1589 |
| 12 | (partial* adj4 (diet* or formula)).mp. | 2078 |
| 13 | ((polymeric or elemental) adj3 diet).mp. | 4034 |
| 14 | (diet* adj2 (intervention or therapy)).mp. | 66754 |
| 15 | 8 or 9 or 10 or 11 or 12 or 13 or 14 | 398133 |
| 16 | Crohn Disease/ | 92239 |
| 17 | Inflammatory Bowel Diseases/ | 13614 |
| 18 | Crohn*.mp. | 106837 |
| 19 | 16 or 17 or 18 | 116616 |
| **20** | **7 and 15 and 19** | **1781** |

| **Overview:** | | |
| --- | --- | --- |
| Interface: EBSCO Database: **Cumulated Index to Nursing and Allied Health Literature (CINAHL)** Search Date: Feb 03, 2021 Study types: No limit on search Publication date: No limit on search | | |
| **Search Strategy:** | | |
| **#** | **Searches** | **Results** |
| 1 | (MH "Child, Preschool") | 212,908 |
| 2 | (MH "Child+") | 680,015 |
| 3 | TX (pediatric* or paediatric*) | 519,367 |
| 4 | TX (child* or adolescen* or teen* or youth*) | 1,326,281 |
| 5 | S1 OR S2 OR S3 OR S4 | 1,417,607 |
| 6 | (MH "Enteral Nutrition") | 9,796 |
| 7 | (MH "Diet Therapy+") | 33,221 |
| 8 | TX Enteral nutrition | 14,468 |
| 9 | TX (exclusive* N3 (diet or formula)) | 427 |
| 10 | TX (partial* N3 (diet or formula)) | 307 |
| 11 | TX ((polymeric or elemental) N3 (diet) | 182 |
| 12 | TX (diet*) N2 (intervention or therapy) | 30,305 |
| 13 | S6 or S7 OR S8 OR S9 OR S10 OR S11 OR S12 | 68,095 |
| 14 | (MH "Crohn Disease") | 6,979 |
| 15 | (MH "Inflammatory Bowel Diseases") | 7,042 |
| 16 | TX (Crohn*) | 9,861 |
| 17 | S14 OR S15 OR S16 | 14,688 |
| **18** | **S5 AND S13 AND S17** | **311** |

| **Overview:** | | |
| --- | --- | --- |
| Interface: Cochrane Library Database: **Cochrane Central Register of Controlled Trials (CENTRAL)** Search Date: Feb 03, 2021 Study types: No limit on search Publication date: No limit on search | | |
| **Search Strategy:** | | |
| **#** | **Searches** | **Results** |
| 1 | MeSH descriptor: [Pediatrics] explode all trees | 678 |
| 2 | MeSH descriptor: [Child] explode all trees | 56347 |
| 3 | MeSH descriptor: [Adolescent] this term only | 104274 |
| 4 | (pediatric* or paediatric*) | 73530 |
| 5 | (child* or adolescen* or teen* or youth*) | 270650 |
| 6 | #1 or #2 or #3 or #4 or #5 | 286814 |
| 7 | MeSH descriptor: [Enteral Nutrition] this term only | 1851 |
| 8 | MeSH descriptor: [Diet Therapy] explode all trees | 5916 |
| 9 | (exclusive* NEAR/4 (diet or formula)) | 265 |
| 10 | (partial* NEAR/4 (diet or formula)) | 304 |
| 11 | (polymeric or elemental) NEAR/3 (diet) | 292 |
| 12 | enteral nutrition | 5937 |
| 13 | (diet*) NEAR/2 (intervention or therapy) | 17685 |
| 14 | #7 or #8 or #9 or #10 or #11 or #12 or #13 | 26179 |
| 15 | MeSH descriptor: [Crohn Disease] this term only | 1612 |
| 16 | MeSH descriptor: [Inflammatory Bowel Diseases] this term only | 559 |
| 17 | Crohn* | 5437 |
| 18 | #15 or #16 or #17 | 5750 |
| **19** | **#6 and #14 and #18** | **169** |

| **Overview:** | |
| --- | --- |
| Interface: U.S. National Library of Medicine Database: Clinicaltrials.gov Search Date: Feb 03, 2021 Study types: No limit on search Publication date: No limit on search | |
| **Search Strategy:** | |
| **Terms** | **Results** |
| (partial enteral nutrition OR exclusive enteral nutrition OR polymeric diet OR elemental diet OR semi-elemental diet) | **174** |

**Supplementary Table 2** List of important excluded studies

| **First Author** | **Year of Publication** | **Reason for Exclusion** |
| --- | --- | --- |
| Sanderson^(1)^ | 1987 | The remission criteria were not clearly defined and the number of patients with remission was not specified |
| Basseri^(2)^ | 2016 | Abstract only: Conference abstract of the same study as Connors 2017 included in the review. |
| Johnson^(3)^ | 2006 | Wrong comparator. The aim of this study was to compare partial enteral nutrition with conventional EEN in active CD. |
| L. Gailhoustet^(4)^ | 2002 | Wrong study design: This is a cross-sectional study involving a quality of life study where the EEN therapy was 40-120 days (beyond 4-8 weeks). |
| Humphrey C^(5)^ | 2019 | Abstract only: health-related quality of life impact of steroids vs. exclusive enteral nutrition. |
| Lee^(6)^ | 2015 | Wrong comparator: this prospective study in children initiating PEN, EEN, or anti-TNF therapy for Crohn's disease compared clinical outcomes and quality of life. No corticosteroid was involved. |
| Hart^(7)^ | 2018 | Abstract only: measuring the quality of life in paediatric CD patients receiving exclusive enteral nutrition or corticosteroids. |
| Levine^(8)^ | 2019 | Wrong comparator: the study compared EEN with the CD exclusion diet, not corticosteroids. |

**References**

1. Sanderson IR, Udeen S, Davies PS *et al.* (1987) Remission induced by an elemental diet in small bowel Crohn's disease. *Arch Dis Child* **62**, 123-127.

2. Basseri SC, J.; Grant, A.; MacIntyre, B.; Giffin, N.; Mahdi, G.; Noble, A.; Rashid, M.; Otley, A. R.; Van Limbergen, J. (2016) Long-term avoidance of steroids associated with exclusive enteral nutrition as induction therapy in paediatric Crohn's disease does not increase surgery, hospitalisations or biologics use: Results of a propensity score-matched cohort analysis. *Journal of Crohn's and Colitis* **10** S64.

3. Johnson T, Macdonald S, Hill SM *et al.* (2006) Treatment of active Crohn's disease in children using partial enteral nutrition with liquid formula: a randomised controlled trial. *Gut* **55**, 356-361.

4. Gailhoustet L, Goulet O, Cachin N *et al.* (2002) [Study of psychological repercussions of 2 modes of treatment of adolescents with Crohn's disease]. *Archives de pediatrie : organe officiel de la Societe francaise de pediatrie* **9**, 110-116.

5. Humphrey C, Grant AK, Walters T *et al.* (2019) A260 HEALTH-RELATED QUALITY OF LIFE IMPACT OF STEROIDS VS. EXCLUSIVE ENTERAL NUTRITION FOR INDUCTION IN A LARGE CANADIAN PEDIATRIC IBD INCEPTION COHORT. *Journal of the Canadian Association of Gastroenterology* **2**, 510-511.

6. Lee D, Baldassano RN, Otley AR *et al.* (2015) Comparative Effectiveness of Nutritional and Biological Therapy in North American Children with Active Crohn's Disease. *Inflamm Bowel Dis* **21**, 1786-1793.

7. Hart L, Farbod Y, Halgren CR *et al.* (2018) A153 MEASURING QUALITY OF LIFE AND DISEASE ACTIVITY IN PEDIATRIC PATIENTS RECEIVING INDUCTION THERAPY OF EXCLUSIVE ENTERAL NUTRITION OR CORTICOSTEROIDS FOR ACTIVE INFLAMMATORY BOWEL DISEASE. *Journal of the Canadian Association of Gastroenterology* **1**, 264-265.

8. Levine A, Wine E, Assa A *et al.* (2019) Crohn's Disease Exclusion Diet Plus Partial Enteral Nutrition Induces Sustained Remission in a Randomized Controlled Trial. *Gastroenterology* **157**, 440-450 e448.
